# Biochemically Programmable Isothermal PCR

**DOI:** 10.1002/advs.202404688

**Published:** 2024-09-13

**Authors:** MinGin Kim, Vijay Ravisankar, Yassin A. Hassan, Victor M. Ugaz

**Affiliations:** ^1^ Artie McFerrin Department of Chemical Engineering Texas A&M University College Station TX 77843 USA; ^2^ Department of Nuclear Engineering Texas A&M University College Station TX 77843 USA

**Keywords:** biochemistry, nucleic acid analysis, PCR, point of care diagnostics

## Abstract

Isothermal PCR can be performed by imposing a static temperature gradient that continuously circulates reagents through denaturing, annealing, and extension conditions inside a PCR tube. But despite early promise, these systems have yet to demonstrate performance and repeatability sufficient for adoption in validated laboratory tests because the rate‐limiting extension step is inherently short and cannot be increased independently of the other stages in a temperature cycle. Here, a discovery that enables isothermal PCR to be achieved with statistically robust repeatability that meets or exceeds diagnostic assay requirements (false positive/negative rate <8% at 95% confidence) by manipulating the interplay between the DNA replication biochemistry (via the amplicon GC content) and the microscale circulatory flow inside a PCR tube is reported. Surprisingly, optimal performance depends on selecting primer sequences that replicate high GC content amplicons, contradicting established PCR primer design rules. This innovative thermocycling approach accelerates PCR to speeds rivaling ultra‐fast instruments, enabling rapid, repeatable isothermal DNA analysis across a range of targets relevant to diagnostics and pathogen detection.

## Graphical abstract

Rapid isothermal PCR is achieved with 100% repeatability by manipulating the interplay between the reaction's biochemistry (via the amplicon GC content) and the lava‐lamp‐like microscale flow established inside a PCR tube whose upper and lower surfaces are isothermally maintained at annealing and denaturing temperatures.

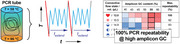

## 1 Introduction

The PCR and its variants (e.g., reverse transcription PCR (RT‐PCR), quantitative real‐time PCR) are analytical gold standards in laboratory‐based molecular biology and diagnostics.^[^ ^1^, ^2^, ^3^, ^4^, ^5^, ^6^ ^]^ This ubiquity has motivated numerous efforts to develop portable PCR instruments, primarily by adopting strategies that miniaturize the system so that batteries can supply sufficient electrical power either to dynamically heat and cool the static reagents or to mechanically pump them through spatially separated static temperature zones during thermocycling.^[^ ^7^, ^8^ ^]^ But miniaturization to nanoliter sample volumes greatly magnifies surface adsorption and evaporation, introducing new challenges and sources of variability. Alternatives to PCR that can be performed isothermally, such as loop‐mediated isothermal amplification (LAMP), have made strides toward overcoming portability barriers.^[^ ^9^, ^10^, ^11^, ^12^, ^13^ ^]^ But while LAMP‐based analysis has shown incredible promise in specific applications, widespread adoption is limited by primer design complexities that, if not optimized, yield reduced sensitivity, specificity, and multiplex capability compared to PCR.^[^ ^14^ ^]^

Thermocycling via Rayleigh–Bénard convection (RB‐PCR) presents a compelling solution by performing PCR in a simplified format that is isothermal with respect to the instrument (i.e., the thermocycling apparatus is held at a constant temperature instead of being repeatedly heated and cooled), achieving rapid amplification while minimizing electrical power consumption.^[^ ^4^, ^5^, ^6^, ^15^, ^16^, ^17^, ^18^, ^19^, ^20^, ^21^ ^]^ Harnessing the same operating principle as a lava lamp, a static temperature gradient applied across a PCR tube generates an internal microscale convective flow that continuously circulates reagents through the replication temperature zones. Unlike prior approaches based on miniaturization, convective thermocycling offers the benefits of an isothermal instrument format to be attained at scales directly compatible with conventional laboratory PCR systems and protocols (e.g., reagent volume 5–30 µL), eliminating the detrimental adsorption and evaporation effects encountered at sub‐microliter scales.

Despite these advantages, isothermal convective PCR has not been widely adopted because, unlike conventional thermocyclers that allow denaturing, annealing, and extension times to be independently programmed, cycling times in RB‐PCR are dictated by the combined action of the velocity and temperature fields comprising the convective flow inside the PCR tube. Consequently, optimization to date has focused on PCR tube designs that minimize the convective flow velocity to permit sufficient extension time, albeit at the expense of prolonging the entire temperature cycle.^[^ ^22^ ^]^ But even these optimal designs only provide a narrow range of achievable transit times through the extension temperature zone under realistic PCR conditions, limiting RB‐PCR to short amplicons. Here, we report a discovery that enables the extension time in convective thermocycling to be biochemically programmed by modulating the thermodynamics governing DNA denaturing and annealing, thereby overcoming a critical limitation on RB‐PCR performance. Unlike prior approaches focused solely on physical system parameters, we show how manipulating the amplicon GC content while simultaneously imposing specific convective flow dynamics enables extension times to be significantly prolonged independent of the other stages in a temperature cycle. We apply this approach to achieve accelerated reaction speeds with 100% repeatability across multiple diagnostically relevant PCR assays. This breakthrough establishes optimal design rules for isothermal convective thermocycling systems with high portability potential, supporting the validation and routine adoption of PCR‐based diagnostics at the point of need.

## 2 Results and Discussion

We identified the most favorable conditions for biochemically programmed thermocycling by performing a series of PCR amplifications using a *λ*‐phage DNA template‐based system that enabled the selection of six different primer sets replicating amplicons at a constant nominal length of 330 bp but with GC content ranging from 40 to 60% (see Table S1, Supporting Information for primer set data; template copy numbers were of order 10^6^). Reactions were performed in ten cylindrical convective PCR tube geometries of varying height‐to‐diameter ratio (*h*/*d*).

We assessed repeatability by adopting an experimental design consisting of at least 36 replicates for each condition tested across the aforementioned six primer sets and ten PCR tube designs, resulting in over 2000 individual replicates. This multi‐dimensional approach (i.e., testing across different primer sets and tube designs) allowed us to broadly evaluate repeatability under conditions related to both the system's physical design and biochemical composition. Considering a zero acceptance number sampling approach, as described by the U.S. Food and Drug Administration (FDA),^[^ ^24^ ^]^ 36 replicates with no failures correspond to repeatability with a false positive/negative rate of <10% at 97% confidence, <8% at 95% confidence, or <6% at 90% confidence. While specific guidelines vary depending on the field of molecular testing, our choice of 36 replicates is consistent with generally established statistical principles for demonstrating repeatability in validated diagnostic assays.^[^ ^25^, ^26^ ^]^

**Figure 1:**
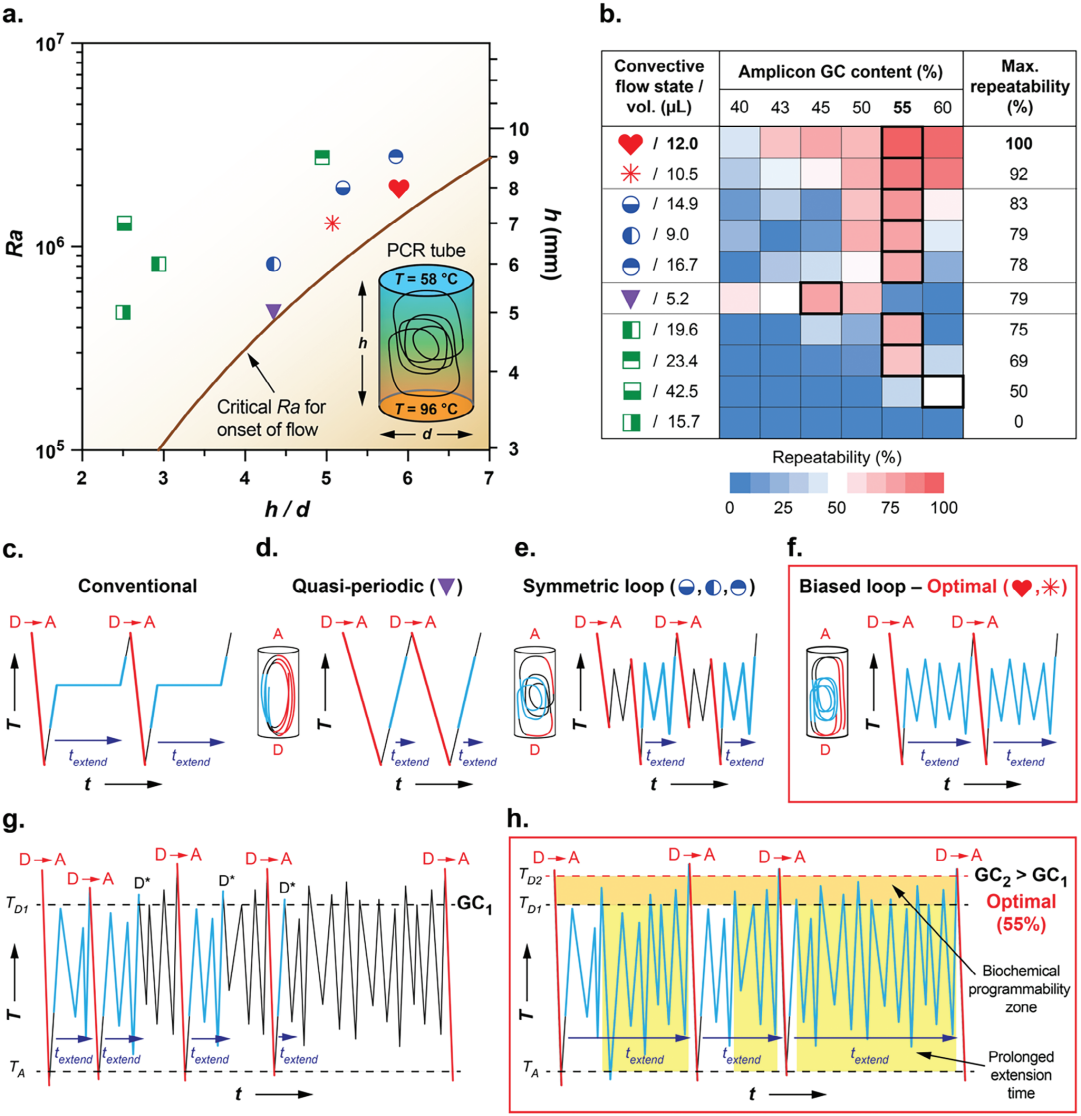
High‐GC amplicons counterintuitively maximize isothermal convective PCR repeatability. a) The ten cylindrical PCR tube geometries studied are displayed on a parametric map. The strength of the convective flow field in PCR tubes with height *h* and diameter *d* is expressed in terms of the dimensionless Rayleigh number, *Ra* (the solid line represents the critical *Ra* for the onset of flow).^[^ ^23^ ^]^ b) Repeatability after performing 36 PCR replicates in each geometry using six different primer sets over a range of amplicon GC content with a nominal target length of 330 bp is depicted in a heat map. The maximum repeatability achieved is indicated in the right‐hand column, and a bold border indicates the corresponding amplicon GC content associated with maximum repeatability. c,d) Temperature versus time associated with c) a conventional PCR temperature cycle and convective d) quasi‐periodic, e) symmetric loop, and f) biased loop trajectories. The optimal biased loop trajectory most closely approaches the conventional profile in (c) by maximizing the frequency of D→A transits and the extension time *t_extend_*. g,h) Increasing the amplicon GC content from a level of g) GC_1_ to h) GC_2_ significantly prolongs *t_extend_* (yellow‐shaded region) by preventing premature denaturing events D* that would otherwise have terminated the temperature cycle. The optimal GC content of 55% maximizes the biochemical programmability zone available to prolong *t_extend_* (orange‐shaded region). The red and blue colored line segments in (c–h) represent stages of the temperature cycle (i.e., the denaturing to annealing transit (red) and extension (blue) stages) but do not imply a constant temperature within each stage.

**Figure** **1**a displays the ten PCR tube configurations tested on a parametric map that plots the strength of the convective flow (quantified by the dimensionless Rayleigh number, *Ra*) versus geometry (quantified by *h/d*). Here, $Ra\;=\;g\beta \Delta Th^{3}/\left(\upsilon \alpha \right)$, where *g* = 9.81 m s^−2^ is the gravitational acceleration, β  =  6.242  ×  10^−4^ °C^−1^ is the fluid's thermal expansion coefficient, Δ*T* =  38 °C is the temperature difference between the upper (annealing, 58 °C) and lower (denaturing, 96 °C) surfaces, *h* is the height of the cylindrical PCR tube, $\upsilon \;=\;3.7486\;\times \;10^{-7}\;m^{2}\;s^{-1}$ is the fluid's kinematic viscosity, and α  =  1.6354  ×  10^−7^ m^2^ s^−1^ is the fluid's thermal diffusivity (see Table S2, Supporting Information, all properties are based on water at 77 °C).

The repeatability observed when an ensemble of 36 PCR replicates was performed in each geometry is shown as a function of amplicon GC content on the corresponding heat map (Figure 1b). Surprisingly, these results point to one PCR tube geometry (*h* = 8.0 mm, *d* = 1.38 mm, *h/d* = 5.8, volume = 12.0 µL; denoted by the red heart symbol) that yields 100% repeatability at an amplicon GC content of 55%. This geometry also delivers the highest repeatability values across the entire range of amplicon GC content. The data further reveal that amplicons with 55% GC content produce the highest repeatability across nearly all PCR tube geometries. Notably, 55% amplicon GC content is considerably higher than the sub‐45% GC value recommended in conventional PCR primer design protocols.^[^ ^27^ ^]^

These data illustrate that RB‐PCR thermocycling performance critically depends on selecting conditions that simultaneously optimize the flow field (governed by *Ra* and *h/d*) and the reaction biochemistry (governed by amplicon GC content). To understand this interplay and identify the unique attributes of the lone PCR tube geometry that achieved 100% repeatability, we obtained thermal profiles representing the temperature that reagents experience as a function of time while circulating through the PCR tube. Computational fluid dynamics simulations were performed to determine the 3‐D flow fields within each of the ten PCR tube geometries in the parametric map of Figure 1a. We then identified 300 randomly distributed trajectories within the flow fields at each geometry and computed the temperature as a function of time following each of them for 300 s.

A benchmark conventional PCR temperature cycle (Figure 1c) is characterized by a rapid transit from denaturing to annealing temperatures (D→A), followed by heating to the extension zone where the temperature is held constant for some time (*t_extend_*) before returning to denaturing conditions. Based on these characteristics, we define optimal convective flows as those that expose reagents to thermal histories that mimic the benchmark thermocycling profile by simultaneously 1) maximizing the number of direct denaturing‐to‐annealing transits and 2) prolonging the residence time at the extension temperature.

Our simulations reveal three primary categories of internal convective flow phenomena depending on the PCR tube geometry. One class of flows is characterized by quasi‐periodic trajectories that circulate reagents between denaturing (bottom surface) and annealing (top surface) temperatures, following paths approximating closed loops (Figure 1d). These trajectories expose reagents to thermal histories that provide numerous direct D→A transits. But the subsequent A→D transits are also direct, limiting *t_extend_*. Additionally, unlike a conventional thermocycler, the closed‐loop nature of these quasi‐periodic flows exposes reagents to thermal profiles that can vary greatly depending on their location within the PCR tube. Consequently, while flows embedding quasi‐periodic flow trajectories have been considered the most desirable design condition in previous adaptations of RB‐PCR,^[^ ^22^ ^]^ optimization is challenging because the residence time in the extension temperature zone is limited and cannot be independently prolonged (i.e., reducing the flow velocity to increase *t_extend_* lengthens the entire temperature cycle).

A second class of convective flows generates trajectories characterized by loops around the center of the PCR tube that occur during transits between denaturing and annealing temperatures (Figure 1e). These central loops enable reagents to be exposed to a longer *t_extend_* than the quasi‐periodic trajectories. But this beneficial effect is limited because the central loops are symmetric, occurring with equal frequency during both the D→A and A→D transits. The resulting interruption in D→A transits constrains *t_extend_* and produces thermal cycling profiles that differ from the benchmark in Figure 1c.

A third class of convective flows is also characterized by loops around the center of the PCR tube (Figure 1f). But these loops are biased such that they occur more frequently as reagents travel from annealing to denaturing temperatures, maximizing the number of direct D→A transits while prolonging *t_extend_*. Consequently, unlike the quasi‐periodic or symmetric loop cases, biased loop trajectories mimic the benchmark temperature cycle by exposing reagents to thermal histories analogous to a conventional three‐step thermocycling protocol. Our simulation data reveal that this biased loop flow state uniquely occurs in the PCR tube geometries displaying the highest repeatability (i.e., the red data points in Figure 1a).

We can now understand the role of amplicon GC content in governing repeatability by focusing on the characteristics of the optimal biased loop flow state. When PCR amplicons with sub‐45% GC content considered optimal in conventional PCR protocols follow trajectories associated with loops around the center of the PCR tube, they experience denaturation upon exceeding a threshold temperature (Figure 1g). But unless reagents subsequently return to the annealing temperature zone, these premature denaturation events (D*) limit *t_extend_* by terminating extension and delaying initiation of the subsequent cycle until the next direct D→A transit. When the amplicon's GC content is increased, the corresponding increase in its melting temperature suppresses premature denaturation, considerably broadening *t_extend_* to occupy the entire time between successive direct D→A transits (the yellow‐shaded region in Figure 1h). Therefore, unlike prior approaches that increase *t_extend_* by reducing the flow velocity, manipulating the amplicon GC content makes it possible to biochemically program the temperature cycle by preventing premature denaturation that would otherwise terminate the cycle, thereby prolonging the extension step without increasing the total cycle time. Optimal biochemical programmability is achieved at 55% amplicon GC content because this condition maximizes the number of premature denaturing events suppressed between successive direct D→A transits (the orange‐shaded region in Figure 1h).

**Figure 2:**
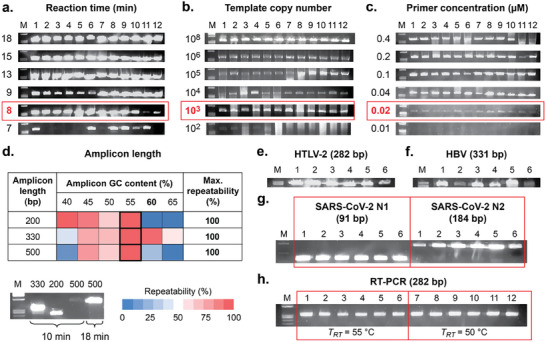
Isothermal convective PCR enables rapid, repeatable diagnostics and pathogen detection. All reactions were performed using the optimal PCR tube geometry (*h* = 8.0 mm, *d* = 1.38 mm, *h/d* = 5.8, volume = 12.0 µL; denoted by 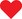 in Figure 1a) and the primer set replicating the optimal amplicon GC content of 55% via a *λ*‐phage DNA template (see Tables S1,S3, Supporting Information for primer set and PCR protocol data, respectively). Replication is achieved with high a) speed and b) sensitivity at c) lower reagent concentrations than conventional protocols (18 min reaction time in (b) and (c)). d) A wide range of amplicon lengths can be replicated (see Tables S4,S5, Supporting Information for primer set data). Optimized conditions allow rapid replication of a range of pathogenic markers, including e) HTLV‐2, f) HBV, and g) SARS‐CoV‐2 from plasmid templates (see Tables S6,S7, Supporting Information for primer set and plasmid template data, respectively). Reaction times of 18 min were employed in (b,c) and (e–g). h) Repeatable RT‐PCR is achievable by incorporating a 10 min isothermal reverse transcription step at *T_RT_* prior to the 18 min PCR replication. Reverse transcription was performed at both 55 °C (lanes 1–6) and 50 °C (lanes 7–12) to demonstrate robustness (see Table S9, Supporting Information for primer set data).

Simultaneous optimization of the convective flow field and the amplicon GC content makes isothermal convective PCR thermocycling highly robust, enabling rapid analysis as demonstrated by replicating a 368 bp target with 100% repeatability in as little as 8 min (**Figure** **2**a, detection at earlier stages of replication is possible in as little as 5 min). We quantified detection limits by serially diluting the DNA template over a range from 10^8^ to 10^1^ copies, achieving 100% repeatability at initial copy numbers as low as 10^3^ (Figure 2b, the target amplicon was replicated at copy numbers as low as 10^1^ at reduced repeatability). The flow field established during convective thermocycling introduces a mixing effect that enhances the reaction kinetics, as evident by our finding that 100% repeatability is achievable at primer concentrations as low as 0.02 mm, tenfold less than in a conventional thermocycler and orders of magnitude lower than other rapid PCR approaches that require elevated reagent concentrations to compensate for faster cycling times (Figure 2c). The optimal GC content of 55% identified in Figure 1 also enables amplicons ranging from 200 to 500 bp in length to be replicated within 100% repeatability (Figure 2d). We applied the optimal PCR tube and primer design conditions described above to replicate targets related to pathogenic markers relevant to point‐of‐need diagnostic applications from plasmid DNA templates, including human T‐cell leukemia virus II (HTLV‐2, Figure 2e), hepatitis B (HBV, Figure 2f), and COVID‐19 (SARS‐CoV‐2, Figure 2g) with 100% repeatability. Finally, we demonstrated repeatable reverse‐transcription PCR from a wild‐type GFP mRNA template by incorporating a 10 min reverse‐transcription incubation step, after which the upper and lower surfaces of the PCR tube were heated to annealing and denaturing temperatures, respectively, for convective thermocycling (Figure 2h). These data demonstrate that biochemically programmable thermocycling delivers a simplified platform for reliable rapid isothermal convective PCR.

It is challenging to directly compare PCR efficiency between traditional and convective PCR formats owing to the different nature of reagent temperature exposure in each method. In convective PCR, a ‘cycle’ exists only in an averaged sense, as reagents continuously follow different local trajectories, whereas, in traditional PCR, the entire reagent volume experiences the same temperature states in a series of discrete steps. While conventional metrics of PCR efficiency are based on discrete thermocycling, we can infer the efficiency of isothermal convective PCR by recalling our prior comparison of real‐time amplification in convective PCR versus a commercial benchtop instrument, which yielded virtually identical standard curves.^[^ ^16^ ^]^ Those previous results were obtained using the same lambda phage DNA template and the primer set replicating the 43% GC amplicon in our present study. Given that our present work now shows the PCR tube design and primer set used in our previous study to be sub‐optimal, it is reasonable to conclude that the previously demonstrated efficiency metrics can be considered a minimum benchmark for the present work. This suggests the potential for even greater efficiency improvements in future optimizations of our convective PCR system, consistent with the ability to maintain performance at lower reagent concentrations than conventional protocols (Figure 2c).

**Figure 3:**
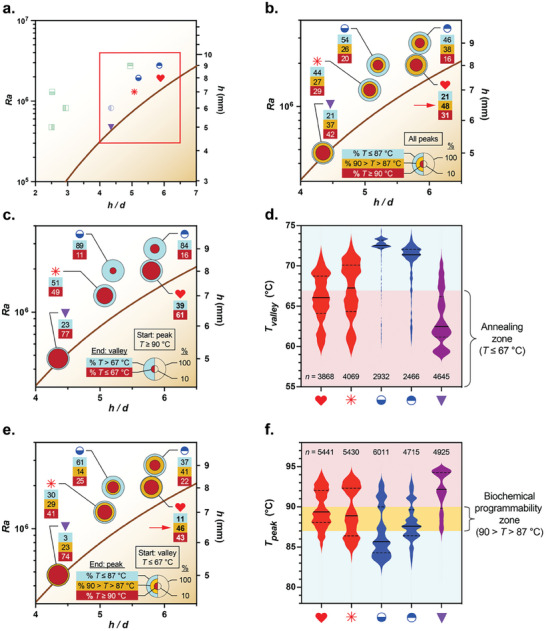
Biochemically programming cycling times in isothermal convective PCR. a) An ensemble of 300 randomly distributed trajectories was analyzed over a period of 300 s using computational fluid dynamics for each of the five PCR tube geometries located within the red box. b) Temperature distribution of all peaks during the 300 s simulation period. c,d) Denaturing‐to‐annealing (D→A) transits are quantified by the c) fraction and d) distribution of the minimum temperature achieved immediately following peak temperatures above 90 °C (*T_valley_*; *n*, the total number of minima across the entire ensemble of 300 trajectories). e,f) Biochemical programmability potential is quantified by the e) fraction and f) distribution of the maximum temperature achieved immediately following valley temperatures below 67 °C (*T_peak_*; *n*, the total number of peaks across the entire ensemble of 300 trajectories). Solid lines in the violin plots in (d) and (f) denote median values; dashed lines indicate 25% and 75% quartiles.

We assessed biochemical programmability by quantifying the attributes of convective flow fields corresponding to five PCR tube geometries that yielded the highest repeatability (**Figure** **3**a). First, quantifying the temperature distribution of all peaks recorded during the 300 s simulation period reveals that the optimal PCR tube geometry displays the highest percentage of peaks in the biochemical programmability zone between 87 and 90 °C (red arrow, Figure 3b). This result supports our observation that the highest repeatability is obtained in biased loop convective flows that generate trajectories with the largest fraction of peaks in this programmability zone (Figure 2h). Second, we examined the value of *T_valley_*, defined as the minimum temperature reached immediately after attaining a peak value above 90 °C. Biased loop and quasi‐periodic flow trajectories maximize the percentage of D→A transits (represented by those generating *T_valley_* ≤ 67 °C, Figure 3c) and minimize *T_valley_* (Figure 3d), consistent with more frequent annealing events that are necessary precursors for successful extension. Third, we quantified *T_peak_*, defined as the maximum temperature reached immediately after attaining a minimum value below 67 °C. The optimal PCR tube geometry uniquely displays a significantly higher percentage of transits reaching *T_peak_* between 87 and 90 °C (red arrow, Figure 3e), and the *T_peak_* values are symmetrically distributed between the biochemical programmability (90 > *T* > 87 °C) and denaturing (*T* ≥ 90 °C) zones (Figure 3f). These features are indicative of trajectories susceptible to premature denaturation and, therefore, most likely to be impacted by increasing the amplicon GC content, consistent with a greater potential to prolong *t_extend_* by increasing the amplicon GC content.

Our analysis also reveals a much different interplay between the convective flow and the amplicon GC content in quasi‐periodic flows, currently considered optimal for RB‐PCR. On the one hand, quasi‐periodic trajectories embed many of the features that make biased loops favorable for PCR, including frequent attainment of peak temperatures in the *T* > 90 °C and 87 < *T* < 90 °C bands and low values of *T_valley_* (purple data point, Figure 3b–d). But unlike biased loops, direct annealing to denaturing transits are strongly favored in quasi‐periodic trajectories, such that the accompanying lack of trajectories embedding loops around the center of the PCR tube severely constrains *t_extend_* (Figure 3e,f). Our data also shed light on why repeatability in the quasi‐periodic case decreases with increasing amplicon GC content in the same way as in conventional thermocycling protocols (i.e., maximum repeatability observed at 45% GC; Figure 1b). High amplicon GC contents suppress denaturation in quasi‐periodic flows because very few trajectories exist in the biochemically programmable zone, leading to a much different amplicon GC content dependence compared to the biased and symmetric loop cases.

**Figure 4:**
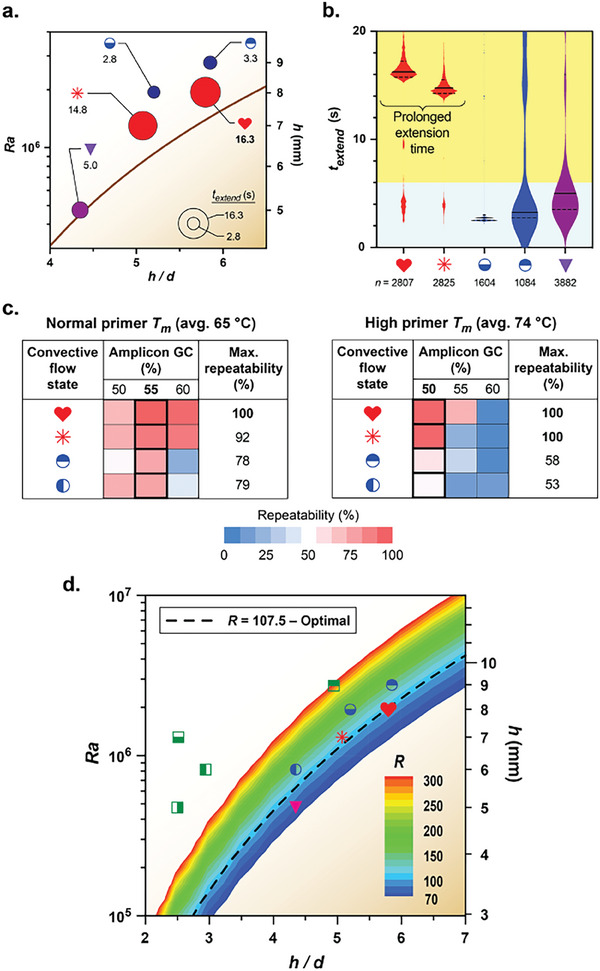
Physico‐chemical optimization maximizes isothermal convective PCR extension times. a,b) An ensemble of 300 randomly distributed trajectories was analyzed over a period of 300 s using computational fluid dynamics for each of the five PCR tube geometries located within the red box in Figure 3a. a) The median and b) distribution of elapsed time, *t_extend_*, between reaching a minimum temperature of *T_valley_* below 67 °C and subsequently attaining a temperature above 90 °C (*n*, total number of flow paths starting from a minimum temperature of *T_valley_* below 67 °C whose next peak is above 90 °C across the entire ensemble of 300 trajectories). c) Increasing the primer *T_m_* from 65 to 74 °C also enables temperature cycling to be biochemically programmed (all reactions were performed using the optimal PCR tube geometry denoted by the red heart symbol in Figure 1; 18 min reaction time; nominal target length of 330 bp; see Table S10, Supporting Information for primer set data). d) The parametric map in Figure 1a is augmented to show the modified Rayleigh number, *R*. The optimal PCR tube geometry denoted by the red heart symbol in Figure 1 corresponds to *R* = 107.5. Plotting this *R*‐value across the entire parameter space (dashed line) establishes a locus of optimal PCR tube geometries that embed dynamically similar flow trajectories that maximize PCR repeatability.

These insights suggest new design rules that harness biochemical programmability to maximize repeatability in isothermal RB‐PCR. First, temperature profiles mimicking a conventional 3‐step cycling protocol are achievable by selecting PCR tube designs that generate biased loop convective flows characterized by loops around the center while simultaneously maximizing the frequency of direct denaturing‐to‐annealing transits (Figure 1f). Second, manipulating the amplicon GC content within the biochemical programmability zone enables the extension time to be prolonged independently of the other stages of the temperature cycle by suppressing premature denaturation events (Figure 1h). We quantified this interplay by extending our flow trajectory analysis to quantify the elapsed time between reaching a minimum temperature of *T_valley_* ≤ 67 °C and subsequently attaining a maximum temperature of *T* ≥ 90 °C, indicative of the achievable extension time *t_extend_* (**Figure** **4**a). This analysis reveals that biased loop trajectories (red data points) yield a fivefold longer median *t_extend_* than symmetric loops (blue data points) and a threefold longer median *t_extend_* than quasi‐periodic flows (purple data point) by simultaneously maximizing the number of D→A transits and the fraction of peaks in the biochemical programmability zone (Figure 4b). These results also highlight why PCR performance is challenging to optimize in quasi‐periodic flows, up to now considered the most desirable for convective thermocycling, because *t_extend_* is constrained and cannot be prolonged independent of the overall cycling time.

In addition to manipulating amplicon GC content, biochemical programmability is also achievable by increasing the primer melting temperatures (*T_m_*) to expand the annealing temperature zone and, to a lesser extent, delay product denaturation. We examined these effects by designing primers to replicate amplicons of similar length and GC content as those employed in our prior PCR repeatability studies (e.g., Figure 1b) but with average *T_m_* increased from 65 to 74 °C (see Table S10, Supporting Information for primer set data). These data show that high‐*T_m_* primers reduce the amplicon GC content associated with maximum repeatability from 55% to 50%, but the maximum repeatability occurs within a significantly narrower window of amplicon GC % (Figure 4c). It is difficult, however, to rely solely on *T_m_* for biochemical programmability because the range of suitable primers becomes constrained as *T_m_* increases. For example, we could not explore amplicon GC contents below 50% because it was impossible to identify target regions in the *λ*‐phage DNA template with GC content high enough to increase primer *T_m_* above 74 °C while simultaneously reducing the amplicon GC content. Therefore, the optimal design of high‐*T_m_* primers becomes challenging because high melting temperatures restrict PCR replication to loci of high‐GC content target sites in the template genome. High primer melting temperatures also increase the likelihood of detrimental effects related to incomplete denaturation, enhancing susceptibility to primer‐dimer and secondary structure formation. Therefore, amplicon GC content offers the most robust pathway to achieve biochemically programmable thermocycling.

Finally, we quantified the combination of physical criteria associated with optimal isothermal convective thermocycling performance using a modified Rayleigh number $R\;=\;g\beta \left(\Delta T/(h/d)\right)d^{3}/\left(16\;\upsilon \alpha \right)$ that embeds the sidewall effects present in PCR tube geometries where *h*/*d* > 1 (Figure 4d).^[^ ^23^ ^]^ Examining the parameters corresponding to the condition where 100% repeatability is observed (*h* = 8.0 mm, *d* = 1.38 mm, *h/d* = 5.8; denoted by the red heart symbol in Figure 1a) yields a value of *R* = 107.5. Plotting an iso‐contour of this *R*‐value across the entire parameter space establishes a locus of optimal PCR tube geometries that embed dynamically similar biased loop flow trajectories with high biochemical programmability potential (dashed line in Figure 4d). Computational simulations across multiple geometries associated with this optimal *R*‐value confirm that the embedded flows display virtually identical biased loop characteristics over a wide range of volumes, suggesting this is a universal design criterion.

## 3 Conclusion

By linking the PCR tube design (governing the physics of microscale convective flow and thermocycling) with the primer composition (regulating PCR biochemistry), biochemical programmability can be exploited to enable rapid and highly repeatable isothermal DNA amplification—a key to equity‐centered deployment of nucleic acid analysis in resource‐limited settings.^[^ ^28^, ^29^ ^]^ These insights also suggest the potential to deliver superior results in applications where conventional PCR is challenging (e.g., GC‐rich amplicons) and, more broadly, reveal new mechanisms capable of mediating spontaneous chemical and biochemical assembly processes in nature.^[^ ^30^ ^]^

## Conflict of Interest

The authors declare no conflict of interest.

## Supporting information



Supporting Information

## Data Availability

The data that support the findings of this study are available from the corresponding author upon reasonable request.
