# Supplementary material for: Biochemically Programmable Isothermal PCR
Source: Adv Sci (Weinh). 2024 Sep 13;11(41):2404688. doi: 10.1002/advs.202404688 (PMC11538674; doi:10.1002/advs.202404688)
Supplement: Supplementary file 1 — Supporting Information [file ADVS-11-2404688-s001.pdf]

ADVANCED SCIENCE
Open Access

Supporting Information

for *Adv. Sci.*, DOI 10.1002/advs.202404688

Biochemically Programmable Isothermal PCR

*MinGin Kim, Vijay Ravisankar, Yassin A. Hassan and Victor M. Ugaz**

Supporting Information

Methods

Apparatus and experiment procedure. Cylindrical PCR tubes were constructed by machining polymethyl methacrylate cubes (3/8-inch tumbled acrylic, Craftics) to heights of 5, 6, 7, 8, and 9 mm. Cylindrical chambers were machined in the cubes to generate the ensemble of ten different geometries in Figure 1 (see Table S2 in the Supporting Information for the dimensions of each PCR tube geometry tested). Microplate sealing tape (PCR-AS-200, Axygen) sealed reagents inside the tubes. A polyethylene film was sandwiched between the tape and the chamber at the bottom (denaturing) surface to prevent bubble formation due to offgassing from the adhesive. The PCR tubes were washed with ddH_2O and treated with Rain-X Anti-Fog (ITW Global Brands), followed by an aqueous solution of 1% bovine serum albumin (Cat. no. A2153; Sigma-Aldrich) for 5 minutes at room temperature to minimize sidewall adsorption and enhance surface wettability. After loading the reagents, PCR was performed by inserting the PCR tube into a heating device programmed to maintain the upper and lower surfaces at 58 and 96 °C, respectively. After incubation for the prescribed reaction time (18 min, unless otherwise noted), the PCR tubes were removed, and the products were analyzed by agarose gel electrophoresis using a Lonza FlashGel system. Repeatability was quantified by the fraction of replicates producing a visible band in the gel. The repeatability data in Figure 1b were obtained by performing 36 replicates for each of the six primer sets at each of the ten *h/d* reactor geometries, generating an ensemble of 2160 replicates across all experimental conditions.

Primer design and PCR conditions. The data in Figures 1b and 2a-d were obtained using a PCR system based on a λ-phage DNA template that enabled the design of six different primer

sets replicating an amplicon with a constant length but with GC content ranging from 40 – 60% (this range was chosen to be centered about the 49.8% average GC content of the λ-phage template DNA). This was accomplished by first identifying regions of the λ-phage DNA template sequence embedding the desired average GC content and then using Primer3 software to generate primer set candidates yielding the desired amplicon length. The candidate primer sets were evaluated according to the following general criteria: (i) 18-24 bp long with 40% to 60% GC content, preferably ending with a purine base (G or C) at the 3' end, and avoiding four or more of a single base (e.g., AAAA) or dinucleotide repeats (e.g., GTGTGT); and (ii) T_m between 50 – 60 °C, with < 5 °C difference between the forward and reverse primers. T_m data were obtained using the Primer-Basic Local Alignment Search Tool (*Primer-BLAST*) provided by the National Center for Biotechnology Information (NCBI).

Stock reaction mixtures contained λ-phage DNA (Cat. no. N3011S, New England Biolabs), forward and reverse primers (Integrated DNA Technologies), and reagents accompanying the KOD DNA polymerase kit (Cat. No. 71085-3, EMD Millipore Sigma). Primer sequences and detailed reagent formulations for the λ-phage and pathogen-based PCR are provided in Tables S3 – S10 of the Supporting Information. Reverse transcription PCR was performed using the qScript One-Step RT-qPCR kit (Cat. No. 95057-200, QuantaBio) following the protocol in Table S9 of the Supporting Information.

Computational fluid dynamics simulations. The 3D flow fields within the cylindrical PCR tubes were simulated using STAR-CCM+ software (Siemens) by simultaneously solving the 3D continuity, Navier-Stokes, and energy conservation equations subject to adiabatic sidewalls and constant temperature boundary conditions of 58 and 96 °C at the upper and lower surfaces,

respectively. The buoyancy-driven force due to the vertical temperature gradient was established using a user-defined field function representing the Boussinesq approximation and included as a momentum source term in the Navier-Stokes equation. The cylindrical simulation domain meshing was also finer (~30 µm) at the boundaries to ensure solution accuracy in the near-wall regions.

The thermal profile analysis in Figures 1a, 3, and 4a-b was performed by releasing a randomly distributed ensemble of 300 Lagrangian massless tracer particles from the mid-horizontal plane of the PCR tube geometry. The position and temperature of each tracer were extracted every 0.25 s over a time period of 300 s. These 1200 data were then post-processed using MATLAB and sorted according to each tracer ID to obtain temperature versus time plots for each of the 300 tracked particles. These temperature profiles were then analyzed to obtain quantitative parameters such as the number of direct denaturing-to-annealing transits, minimum temperature, number of peaks near the denaturing temperature zone, and the extension time, as indicated in Figures 3 and 4a-b.

Table S1. Primer sets used to generate amplicons with variable GC content at a constant **nominal length of 330 bp**. The repeatability studies in Figure 1 involve the primer sets ranging from 40 – 60 nominal amplicon GC%. The amplicon length studies in Figure 2d include the primer set with 65% nominal amplicon GC%. All primers were obtained from Integrated DNA Technologies.

**Amplicon GC% (nominal)**	**Amplicon GC% (actual)**	**Amplicon length (bp)**	**Primer sequence**	**Primer GC%**	**Primer T_m (°C)**
**40**	40.7	329	Fwd: 5' - CCCTAGGACTGCTATGTGCCGGAG - 3'	62.5	66.2
			Rev: 5' - GCAACGTTTCAGCAGCTACAGTCAG - 3'	52.0	64.7
**43**	43.0	237	Fwd: 5' - CTGAGGCCGGGTTATTCTTG - 3'	55.0	58.3
			Rev: 5' - CGACTGGCCAAGATTAGAGA - 3'	50.0	56.7
**45**	44.5	337	Fwd: 5' - GGAGCATCAGGCAGACCCCTCC - 3'	68.2	66.7
			Rev: 5' - GAAGGCGAAGTCATGAGCGCC - 3'	61.9	64.2
**50**	51.5	309	Fwd: 5' - CCGTCTATGACGTGGCTTCCGGC - 3'	65.2	67.6
			Rev: 5' - CAACACGGTTCAGCAACACCCG - 3'	59.1	64.9
**55**	54.0	368	Fwd: 5' - GTAGCCCGTCGGCAGAATCGACC - 3'	65.2	67.3
			Rev: 5' - GCACGATTTAGAGGTCTACCGCCC - 3'	58.3	65.1
**60**	57.8	330	Fwd: 5' - GATGGCTATTCTCAGCGAGCGCC - 3'	60.9	65.9
			Rev: 5' - CGATTTCCCAGAGCACCACGCTG - 3'	60.9	66.2
**65**	64.4	342	Fwd: 5' - GATGGCTATTCTCAGCGAGCGCC - 3'	75.0	70.0
			Rev: 5' - CGATTTCCCAGAGCACCACGCTG - 3'	50.0	60.0

Table S2. Dimensions, volume, Rayleigh number (*Ra*), and modified Rayleigh number (*R*) for each of the ten PCR tube geometries studied.

Symbol in Fig. 1	h(mm)	d (mm)	h/d	Volume (μL)	$Ra = \dfrac{g\beta\Delta T h^3}{\upsilon\alpha}$	$R = \dfrac{\frac{g\beta\Delta T}{(h/d)} d^3}{16\,\upsilon\alpha}$
▼	5.00	1.15	4.35	5.2	474469	83.0
◨	5.00	2.00	2.50	15.7	474469	759.2
◐	6.00	1.38	4.35	9.0	819883	143.4
◧	6.00	2.04	2.94	19.6	819883	684.8
✳	7.00	1.38	5.07	10.5	1301944	122.9
⬓	7.00	2.78	2.52	42.5	1301944	2024.2
♥	8.00	1.38	5.80	12.0	1943426	107.5
◒	8.00	1.54	5.20	14.9	1943426	165.9
◓	9.00	1.54	5.85	16.7	2767105	147.5
⬒	9.00	1.82	4.95	23.4	2767105	289.2

Ra and *R* were calculated using the following property data: $g = 9.81$ m/s^2 is the gravitational acceleration, $\beta = 6.242 \times 10^{-4}$ °C^{-1} is the fluid's thermal expansion coefficient, $\Delta T = 38$ °C is the temperature difference between the denaturing and annealing surfaces, h is the height of the cylindrical PCR tube, $\upsilon = 3.7486 \times 10^{-7}\ \mathrm{m^2/s}$ is the fluid's kinematic viscosity, and $\alpha = 1.6354 \times 10^{-7}\ \mathrm{m^2/s}$ is the fluid's thermal diffusivity (all properties are based on water at 77 °C).

Table S3. Reagents and protocol for the λ-phage template DNA-based convective PCR replication studies corresponding to the data in Figures 1b, 2a-d, and 4c. A 50 μL master mix of each formulation was produced using the KOD DNA polymerase kit (Cat. No. 71085-3, EMD Millipore Sigma).

Reagent	Volume (μL)	Details
Buffer #1 (10x)	5	Supplied with KOD DNA polymerase kit (Cat. No. 71085-3, EMD Millipore Sigma)
$MgCl_2$	2	
dNTP mix	5	
KOD polymerase	0.4	
Forward primer (10 μM)	2	See Tables S1, S3, and S4 for sequence data (Integrated DNA Technologies)
Reverse primer (10 μM)	2	
λ-phage template DNA (0.5 ng/μL)	2	(Cat. no. N3011S, New England Biolabs)
ddH_2O (to 50 μL total volume)	31.6	

Table S4. Primer sets used to generate amplicons with variable GC content at a constant **nominal length of 200 bp** for the amplicon length studies in Figure 2d. All primers were obtained from Integrated DNA Technologies.

Amplicon GC% (nominal)	Amplicon GC% (actual)	Amplicon length (bp)	Primer sequence	Primer GC%	Primer T_m (°C)
**40**	41.2	250	Fwd: 5' - CCCCTCCGTGGATCTGATTCG - 3'	60.0	60.0
			Rev: 5' - CTGACAGTGACAGACTGCGT - 3'	55.0	60.0
**45**	46.2	222	Fwd: 5' - CAAACTGCGCAACTCGTGAA - 3'	50.0	60.0
			Rev: 5' - ATTGCCATCCCAACAGCA - 3'	50.0	57.1
**50**	51.3	226	Fwd: 5' - TATGGCAAAAAGCACCGGGA - 3'	50.0	60.3
			Rev: 5' - GGATGGACTTTGGCCAGACC - 3'	60.0	60.7
**55**	54.7	245	Fwd: 5' - GCTGGCTGACATTTTCGG - 3'	55.6	56.8
			Rev: 5' - GTCGATGGCGTTCGTACTCA - 3'	55.0	60.2
**60**	59.6	203	Fwd: 5' - GCCACCAGTGCCGGTGCGGCGAA - 3'	73.9	74.4
			Rev: 5' - GTTGCCGAGGAAGCTGCACG - 3'	65.0	64.6
**65**	65.6	212	Fwd: 5' - GCGGTGTCCGGCGACAGCCT - 3'	75.0	70.0
			Rev: 5' - TGTTGCAGACGGGCGATAAT - 3'	50.0	60.1

Table S5. Primer sets used to generate amplicons with variable GC content at a constant **nominal length of 500 bp** for the amplicon length studies in Figure 2d. All primers were obtained from Integrated DNA Technologies.

Amplicon GC% (nominal)	Amplicon GC% (actual)	Amplicon length (bp)	Primer sequence	Primer GC%	Primer T_m (°C)
**40**	39.4	477	Fwd: 5' - GTTTTGTTGATGATTTATGTC - 3'	28.6	49.4
			Rev: 5' - CAGTGACAGACTGCGTGTTG - 3'	55.0	59.4
**45**	45.1	459	Fwd: 5' - CAAACTGCGCAACTCGTGAA - 3'	50.0	60.0
			Rev: 5' - ACAACAAACCATGTCGCTGC - 3'	50.0	60.0
**50**	51.3	491	Fwd: 5' - TTCTGCGGTAAGCACGAACT - 3'	50.0	60.0
			Rev: 5' - CATCCGTGGCAATCATGCTG - 3'	55.0	60.0
**55**	54.5	479	Fwd: 5' - GATGCTGCAATTCAGAGCGG - 3'	55.0	60.0
			Rev: 5' - GATGATCTGCGCTACCTGCT - 3'	55.0	60.0
**60**	59.9	486	Fwd: 5' - CGCGGCGGCCACCAGTGCCGG - 3'	85.7	75.6
			Rev: 5' - TCCGCATCCTCAAGCGCGAC - 3'	65	65.6
**65**	64.4	486	Fwd: 5' - GTGCGCTTGCAGGCCAGCTTG - 3'	66.7	67.8
			Rev: 5' - CCATCATGACGCCGATGGAG - 3'	60.0	61.0

Table S6. Primer sets used for replication of pathogen targets in Figure 2e-g. All primers were obtained from Integrated DNA Technologies.

Target	Amplicon GC% (actual)	Amplicon length (bp)	Primer sequence	Primer GC%	Primer T_m (°C)
**HTLV-2**	53.5	282	Fwd: 5' - TGCCAGGGGCTTCTATACCT - 3'	55.0	60.0
			Rev: 5' - TGGCATTGGTGGCTCTTAGG - 3'	55.0	60.0
**HBV**	57.0	331	Fwd: 5' - CAAACTGCGCAACTCGTGAA - 3'	50.0	62.4
			Rev: 5' - ACAACAAACCATGTCGCTGC - 3'	50.0	59.4
**SARS-CoV-2 (N1)**	51.0	91	Fwd: 5' - GGCAGTCAAGCCTCTTCTCG - 3'	60.0	60.7
			Rev: 5' - GAAGTTCCCCTACTGCTGCC - 3'	60.0	60.4
**SARS-CoV-2 (N2)**	51.0	184	Fwd: 5' - ACAATGCTGCAATCGTGCTAC - 3'	74.6	59.9
			Rev: 5' - CCATTGCCAGCCATTCTAGC - 3'	55.0	59.3

Table S7. Plasmid templates used for replication of pathogen targets in Figure 2e-g.

Target	Plasmid template	Template length (bp)	Vendor	Cat no.
**HTLV-2**	HTLV-II, deltaH6H11, in pBR322, purified plasmid DNA	13200	ATCC	45134D
**HBV**	AM6 [EC-AM6, pAM6], purified plasmid DNA	7500	ATCC	45020D
**SARS-CoV-2 (N1, N2)**	pUC57-2019-nCoV-PC:N plasmid	3089	GenScript Biotech, via Molecular Cloud	MC_0101077

Table S8. Reagents and protocol for the plasmid template DNA-based convective PCR replication studies corresponding to the data in Figures 2e-g. A 50 µL master mix of each formulation was produced using the KOD DNA polymerase kit (Cat. No. 71085-3, EMD Millipore Sigma).

**Reagent**	**Volume (µL)**	**Details**
Buffer #1 (10x)	5	Supplied with KOD DNA polymerase kit (Cat. No. 71085-3, EMD Millipore Sigma)
$MgCl_2$	2	
dNTP mix	5	
KOD polymerase	0.4	
Forward primer (10 µM)	2	See Table S5 for sequence data (Integrated DNA Technologies)
Reverse primer (10 µM)	2	
Plasmid DNA (diluted to obtain 0.04 ng/µL final concentration in master mix)	2	See Table S7
ddH_2O (to 50 µL total volume)	31.6	

Table S9. Reagents and protocol for the reverse transcription convective PCR replication studies corresponding to the data in Figure 2h. This reaction generated a 282 bp amplicon with a 40.1% GC content. A 50 µL master mix was produced using the qScript One-Step RT-qPCR kit (Cat. No. 95057-200, QuantaBio).

**Reagent**	**Volume (µL)**	**Details**
One-Step master mix (2x)	25	Supplied with qScript One-Step RT-qPCR kit (Cat. No. 95057-200, QuantaBio)
qScript One-Step reverse transcriptase	1	
Forward primer (10 µM)	2	Fwd: 5' - ATGAGTAAAGGAGAAGAACTTTT - 3'<br>(30.4% GC, T_m = 53.6 °C; Sigma Aldrich)
Reverse primer (10 µM)	2	Rev: 5' - CTGTACATAACCTTCGGGC - 3'<br>(52.6% GC, T_m = 55.4 °C; Sigma Aldrich)
GFP wild type mRNA (717 bp, 46.2 ng/µL)	2	mRNA sequence ordered from Integrated DNA Technologies
ddH_2O (to 50 µL total volume)	18	

Table S10. High T_m primer sets used to generate amplicons with variable GC content at a constant **nominal length of 330 bp** for the studies in Figure 4c.

**Amplicon GC% (nominal)**	**Amplicon GC% (actual)**	**Amplicon length (bp)**	**Primer sequence**	**Primer GC%**	**Primer T_m (°C)**
**50**	51.5	309	Fwd: 5' - GCGACTCACCACGGGCCACGGCT - 3'	73.9	73.4
			Rev: 5' - TTGCCCCCGGCGTCGCGGCACTG - 3'	78.3	76.2
**55**	55.5	328	Fwd: 5' - GCGCGTAGCCCGTCGGCAGAATCG - 3'	70.8	72.1
			Rev: 5' - TGGTTCAGGGATCGCCTCACCACGG - 3'	64.0	70.2
**60**	60.5	296	Fwd: 5' - GCCACCAGTGCCGGTGCGGCGAA - 3'	73.9	74.4
			Rev: 5' - GCCGCGGCAGAGGCGCTCCGTTCC - 3'	79.2	76.0
